# Secondary Metabolites, including a New 5,6-Dihydropyran-2-One, Produced by the Fungus *Diplodia corticola*. Aphicidal Activity of the Main Metabolite, Sphaeropsidin A

**DOI:** 10.3390/molecules27072327

**Published:** 2022-04-04

**Authors:** Maria Michela Salvatore, Ilaria Di Lelio, Marina DellaGreca, Rosario Nicoletti, Francesco Salvatore, Elia Russo, Gennaro Volpe, Andrea Becchimanzi, Alla Eddine Mahamedi, Akila Berraf-Tebbal, Anna Andolfi

**Affiliations:** 1 Department of Chemical Sciences, University of Naples Federico II, 80126 Naples, Italy; mariamichela.salvatore@unina.it (M.M.S.); dellagre@unina.it (M.D.); frsalvat@unina.it (F.S.); 2 Institute for Sustainable Plant Protection, National Research Council, 80055 Portici, Italy; 3 Department of Agriculture, University of Naples Federico II, 80055 Portici, Italy; ilaria.dilelio@unina.it (I.D.L.); rosario.nicoletti@crea.gov.it (R.N.); elia.russo@unina.it (E.R.); gennaro.volpe2@unina.it (G.V.); andrea.becchimanzi@unina.it (A.B.); 4 Research Center for Olive, Fruit, and Citrus Crops, Council for Agricultural Research and Economics, 81100 Caserta, Italy; 5 Department of Biology, Faculty of Natural Sciences, Life and Earth Sciences, University of Ghardaia, Ghardaia 47000, Algeria; aladin1342@yahoo.com; 6 Mendeleum-Institute of Genetics, Faculty of Horticulture, Mendel University in Brno, 69144 Lednice, Czech Republic; berraf.a@hotmail.fr; 7 BAT Center-Interuniversity Center for Studies on Bioinspired Agro-Environmental Technology, University of Naples Federico II, 80055 Portici, Italy

**Keywords:** fungal metabolites, botryosphaeriaceae, metabolomics, natural products, sphaeropsidins

## Abstract

An undescribed 5,6-dihydropyran-2-one, namely diplopyrone C, was isolated and characterized from the cultures of an isolate of the fungus *Diplodia corticola* recovered from *Quercus suber* in Algeria. The structure and relative stereostructure of (5*S*,6S,7*Z*,9*S*,10*S*)-5-hydroxy-6-(2-(3-methyloxiran-2-yl)vinyl)-5,6-dihydro-2H-pyran-2-one were assigned essentially based on NMR and MS data. Furthermore, ten known compounds were isolated and identified in the same cultures. The most abundant product, the tetracyclic pimarane diterpene sphaeropsidin A, was tested for insecticidal effects against the model sucking aphid, *Acyrthosiphon pisum*. Results showed a toxic dose-dependent oral activity of sphaeropsidin A, with an LC_50_ of 9.64 mM.

## 1. Introduction

*Diplodia* (Dothideomycetes, Botryosphaeriaceae) is a widely diffused genus of fungi with more than 1000 described species [1]. Over the years, these species have been reported as pathogens or endophytes of many woody plants [1,2]. Moreover, they represent a prolific source of bioactive products with huge structural variability and bioactivities [3,4,5,6]. In this respect, the capacity of *Diplodia* spp. to have distinct habitus and interactions with plants may be related to the release of bioactive compounds during the spread in host tissues.

Among the *Diplodia* species, *Diplodia corticola* A.J.L. Phillips, A. Alves, and J. Luque is particularly regarded for the production of secondary metabolites [7,8,9,10]. It is frequently associated with dieback and canker diseases of oaks in many Mediterranean countries [11,12,13]. Among the metabolites frequently isolated from in vitro cultures of *D. corticola*, sphaeropsidin A is particularly promising for practical applications in agriculture and medicine due to its exciting biological properties, including antimicrobial, insecticidal, herbicidal, and anticancer activities [14]. Besides potential applications, the documented antimicrobial and insecticidal effects of sphaeropsidin A [15,16,17,18] are relevant for further consideration of the ecological role of the fungus.

In this work, a strain of *D. corticola* isolated from *Quercus suber* in Algeria was investigated in order to increase the available data on the secondary metabolism of this fungus, leading to the isolation of a new 5,6-dihydropyran-2-one, namely diplopyrone C, and ten known compounds which include sphaeropsidin A. Following documented evidence of insecticidal properties [15,18], sphaeropsidin A was tested for aphicidal activity on the pea aphid *Acyrthosiphon pisum* (Harris) (Hemiptera, Aphididae), which is a cosmopolitan polyphagous insect and one of the primary species used as laboratory models for testing the susceptibility of sucking insects to oral administration of insecticidal products by using a feeding bioassay on an artificial diet.

## 2. Results

### 2.1. Secondary Metabolites from Cultures of Diplodia corticola B305

**Figure 1:**
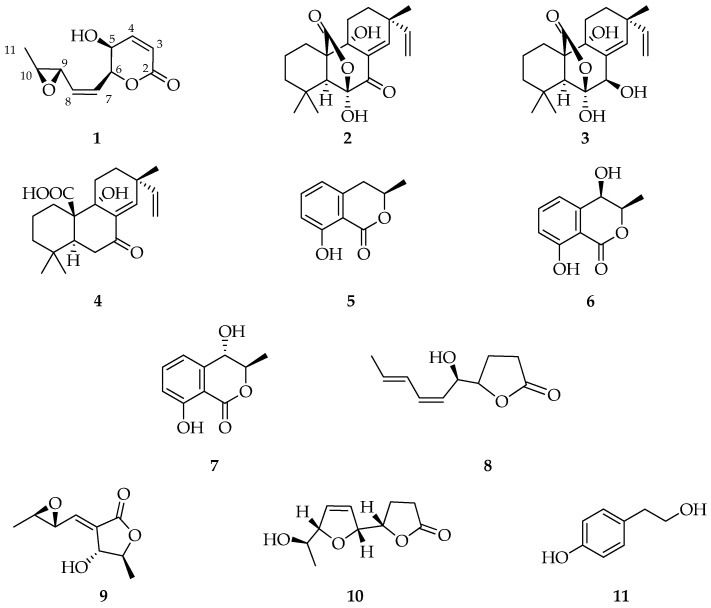
Structures of diplopyrone C (**1**), sphaeropsidins A (**2**), B (**3**), and C (**4**), (3*R*)-mellein (**5**), (3*R*,4*R*)-(**6**), and, (3*R*,4*S*)-4-hydroxymelleins (**7**), sapinofuranone B (**8**), pinofuranoxin A (**9**), diplobifuranylone B (**10**), and tyrosol (**11**).

Crude extract obtained from the culture of *D. corticola* (strain B305), through a chromatographic purification process (see Section 4.3), gave a new metabolite, herein named diplopyrone C (**1**, Figure 1). Its structure was determined by spectroscopic methods, essentially 1D and 2D NMR, IR, and UV combined with mass spectrometry, as reported below (Figures S1–S8). Moreover, ten known metabolites were identified by comparison of their proton spectra (Figures S9–S18), and eventually optical rotation, with those reported in the literature for: sphaeropsidins A and B (**2** and **3**, [19]), sphaeropsidin C (**4**) [20], (*R*)-mellein, (3*R*,4*R*)- and (3*R*,4*S*)-4-hydroxymelleins (**5**–**7**) [21], sapinofuranone B (**8**) [22], pinofuranoxin A (**9**) [23], diplobifuranylone B (**10**) [24], and tyrosol (**11**) [25] (Figure 1).

The HRESI-MS of compound **1** showed picks at *m*/*z* = 235.0395 [M + K]^+^, and 219.1860 [M + Na]^+^ suggesting the molecular formula C_10_H_12_O_4_ and five degrees of unsaturation. Moreover, fragment ion at *m*/*z* = 179.0721 [M-OH]^+^ was evident (Figure S8).

**Figure 2:**
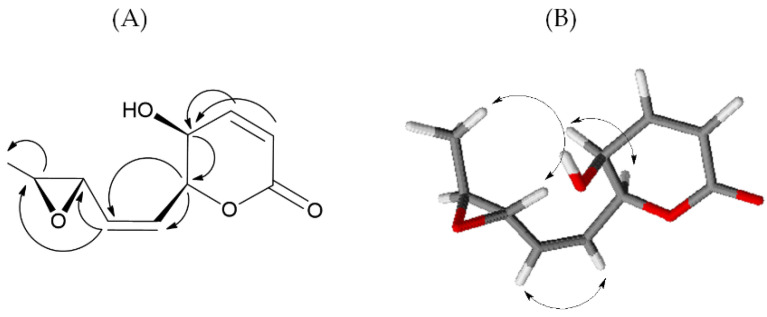
Significant HMBC (**A**) and NOESY (**B**) correlations of **1**.

^1^H and ^13^C NMR analysis showed signals typical of 5,6-disubstitute-5,6-dihydropyran-2-one (Figures S1 and S2) [26] in agreement with IR and UV spectra. In fact, the ^1^H NMR spectrum showed signals at δ (*J* in Hz): 7.03 (dd, 9.8, 5.4), 6.17 (d, 9.8) 5.40 (dd, 7.9, 3.3), and 4.28 (dd, 5.4, 3.3) assigned to H-4, H-3, H-6 and H-5 of 5,6-dihydropyran-2-one ring (Table 1). The COSY spectrum (Figure S3) confirmed this hypothesis, and chemical shifts H-3 to H-6 protons were assigned. In the ^13^C NMR spectrum (Figure S2), signals at δ 162.8, 122.9, 144.3, and 76.9 confirmed the presence of an α,β-unsaturated lactone. Moreover, the signal at δ 4.28 (H-5) correlated to the carbon at δ 63.2 in the HSQC spectrum (Figure S4), indicating the presence of the hydroxyl group at C-5. The remaining signals observed in the 1H NMR spectrum at δ (*J* in Hz): 5.93 (ddd, 11.7, 7.9, 1.1), 5.63 (ddd, 11.7, 6.7, 1.1), 3.34 (dd, 5.2, 2.1 H), 3.00 (dq, 5.2, 2.1 Hz), and 1.40 (d, 5.2) were correlated, in the HSQC spectrum, to δ 127.2, 132.4, 55.5, 56.7, and 17.5, respectively. Analysis of the HMBC spectrum (Figure S5) showed correlations between H-6 and C-7 and C-8, and H-8 with C-9 and C-10 indicated a 3,4-oxirane-1-pentenyl side chain at C-6 (Figure 2).

The *Z* configuration of the double bond at C-7-C-8 was assigned on the basis of the typical coupling constant (11.7 Hz) [27]. The relative configuration of compound **1** was assigned on the basis of the NOE effects observed in the NOESY spectrum (Figure S6). The NOE effect of H-7 with H-8 confirmed the *Z* configuration of the chain double bond. The NOE effect of H-5 with H-6 and the coupling constant of 3.3 Hz indicated a *cis* configuration of the hydroxyl group and the side chain of the dihydropyrone ring. The absence of the NOE effect between the H-9 and the H-10 and the NOE effect of the H-11 methyl with the H-9 indicated a *trans* configuration of the oxirane ring (Figure 2). All spectral data allowed to the determine the structure and relative stereostructure of compound **1** as (5*S*,6S,7*Z*,9*S*,10*S*)-5-hydroxy-6-(2-(3-methyloxiran-2-yl)vinyl)-5,6-dihydro-2H-pyran-2-one, named diplopyrone C.

### 2.2. Oral Toxicity of Sphaeropsidin A on Acyrthosiphon pisum

**Figure 3:**
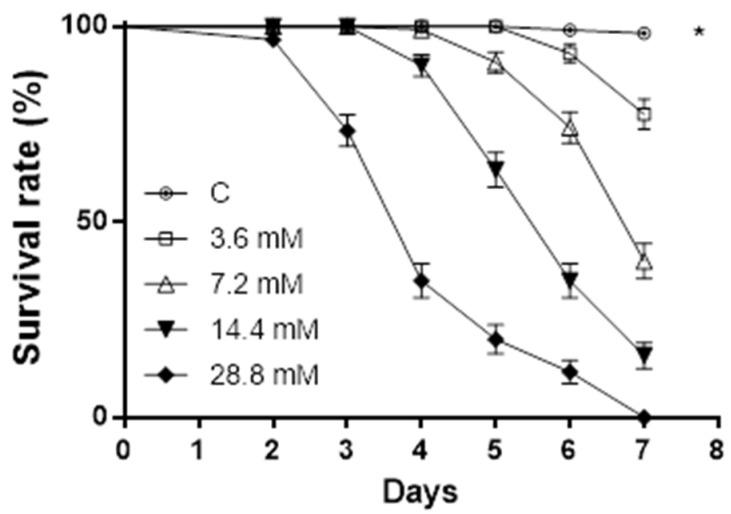
Sphaeropsidin A oral toxicity on *A. pisum*. Aphids’ survival rate was negatively affected by sphaeropsidin A oral administration. Asterisk indicates a statistical difference to log-rank (Mantel–Cox) test (*p* < 0.0001).

Sphaeropsidin A showed an oral lethal activity on aphids at all the doses tested, and the resulting survival rate was significantly lower compared to the control (log-rank test: ꭕ^2^ = 561.1, *p* < 0.0001, dF = 4). The highest doses induced mortality starting from day 2. The mortality increased over time in a dose-dependent manner, and no aphids survived after 7 days of administration (Figure 3).

**Figure 4:**
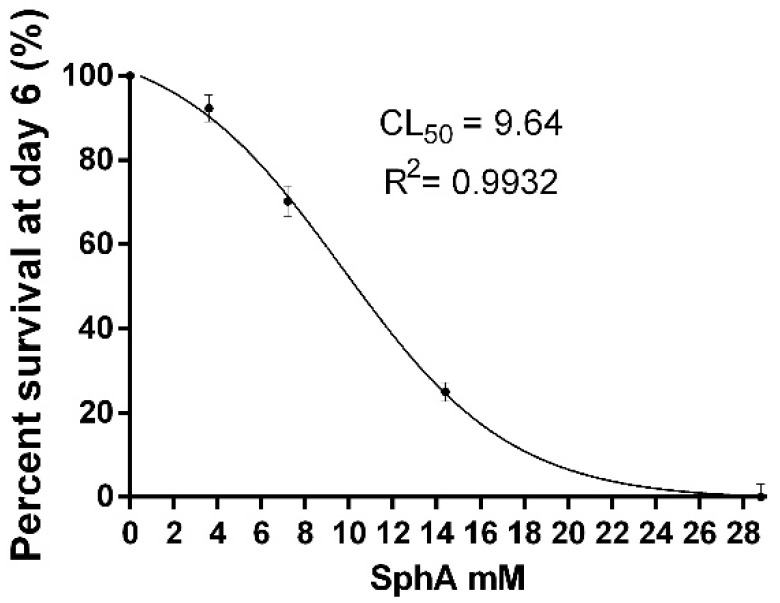
The dose-dependent survival rate of aphids exposed to sphaeropsidin A. Values are reported as mean ± standard deviation (SD) of three replicates in four separate experiments and expressed as a percentage of the control aphids.

The lethal concentrations of sphaeropsidin A, resulting in 10%, 50%, and 90% mortality of the pea aphids (LC_10_, LC_50_, and LC_90_, respectively) and 95% confidence intervals, were determined on day 6 and showed that LC_10_ (95% CI) is 4.5 mM (4.12–4.93), LC_50_ (95% CI) is 9.64 mM (9.23–10.06), and LC_90_ is 20.43 mM (18.81–21.57). The lethal activity of sphaeropsidin A on day 6 is shown in Figure 4.

## 3. Discussion

Secondary metabolites are often used by microbes to enable unique trophic lifestyles, overcome competition with other microbes, or cope with environmental biotic and abiotic stress [28,29,30,31]. Hence, in this study, the production of secondary metabolites from a strain of *D. corticola* was examined with reference to its possible ecological role.

The new 5,6-dihydropyran-2-one, namely diplopyrone C, and several known metabolites were isolated and identified from the fungal strain under examination. This metabolite is closely related to diplopyrone B, which was recently isolated from the same fungal species associated with *Q. suber* in Sardinia (Italy) and characterized as the 5-hydroxy-6-(penta-1,3-dienyl)-5,6-dihydro-pyran-2-one [7]. The 6-substituted derivatives of 5,6-dihydropyran-2-ones (or 5,6-dihydro-α-pyrones) are polyketides produced by several microorganisms and plants. Many of these products are biologically active, exhibiting phytotoxicity, cytotoxicity against tumor cells, and antimicrobial activity [32,33].

Moreover, accurate screening of the existing literature showed that a number of metabolites identified in this study were previously reported as products of *Diplodia* species. In particular, sphaeropsidins A–C (**2**–**4**), (3*R*,4*R*)- and (3*S*,4*R*)-hydroxymelleins (**6**,**7**), sapinofuranone B (**8**), and diplobifuranylone B (**10**) were already identified from cultures of *D. coriticola* (Table 2).

(*R*)-Mellein, (3*R*,4*R*)- and (3*S*,4*R*)-hydroxymelleins (**5**–**7**) belong to the group of 3,4-dihydroisocoumarins, also known as melleins, which are lactonic natural products [39] commonly produced in vitro by botryosphaeraceous fungi, such as *Lasiodiplodia* spp., *Macrophomina* spp., and *Neofusicoccum* spp. [40,41,42]. Furthermore, (*R*)-mellein is considered a vivotoxin since it was also isolated from plants inoculated with the mycelium of *Neofusicoccum parvum* [43].

The high production rate of sphaeropsidin A suggests its possible involvement in the dynamic interaction with the host plant; in this regard, the anti-insectan properties deserve particular attention in regard to the hypothesis that this compound is also released in vivo. In fact, a widespread anti-insectan effect is corroborated by results of previous studies showing fagodeterrent and larvicidal activity against the mosquito *Aedes aegypti* (Diptera, Culicidae) [15] and oral toxic activity against larvae of the chewing model insect *Spodoptera littoralis* (Lepidoptera, Noctuidae) [18].

Here we have demonstrated the possible effects of sphaeropsidin A on sucking insects based on a dose-dependent toxic oral activity against the model phloem sucking insect, *A. pisum*. Hence, if confirmed in planta, production of this secondary metabolite may reduce the impact of herbivorous insects, representing an indication of defensive mutualism established during the development of this fungus as an endophyte or latent pathogen.

Moreover, the oral toxic activity shown by sphaeropsidin A on pea aphids stimulates further investigation of its mode of action from the perspective of its possible application as a new pesticide that meets the growing demand for alternative products with low environmental impact.

## 4. Materials and Methods

### 4.1. General Experimental Procedures

The optical rotations of pure metabolites were measured in CHCl_3_ or MeOH on a Jasco P-1010 digital polarimeter (Tokyo, Japan). FT–IR spectra were recorded in modality ATR (attenuated total reflectance) with model Nicolet 5700 by Thermo Electric Corporation (Waltham, MA, USA). The measuring cell consisted of a mono crystal of zinc selenide. The blank was recorded using air as reference. UV spectra were recorded in CH_3_CN by Cary model 5000 Spectrophotometer by Varian C. (Palo Alto, CA, USA). ^1^H and ^13^C NMR spectra were recorded on a Bruker AMX instrument at 400 and 100 MHz, respectively, in CDCl_3_. The same solvents were used as internal standards. COSY-45, HSQC, HMBC, and NOESY were performed using standard Bruker microprograms. TLC was performed on silica gel (Kieselgel 60, F_254_, 0.25 mm, Merck, Darmstadt, Germany) or reverse-phase plates (Whatman, KC18 F_254_, 0.20 mm). The spots were visualized by exposure to UV radiation (253 nm) or by spraying first with 10% H_2_SO_4_ in methanol followed by heating at 110 °C for 10 min. Chromatography was performed on silica gel column (Merck, Kieselgel 60, 0.063–0.200 mm). HRESI-TOF mass spectra were measured on an Agilent Technologies ESI-TOF 6230DA instrument in the positive ion mode (Milan, Italy).

### 4.2. Fungal Strain and Cultures Production

*Diplodia corticola* strain (B305) employed in this study was previously isolated from *Q. suber* trees showing canker and dieback symptoms in Algeria. The strain was identified and characterized as a pathogen in a previous work [11] based on the integration of morphological features and phylogenetic analysis of the combined ITS and *tef1-α* sequence data. The nucleotide sequences of *D. corticola* are available in GenBank database under accession numbers MT015626 and MT066136.

Liquid cultures of the strain were prepared in Czapek-Dox broth (Oxoid, Thermo Scientific, Waltham, MA, USA) amended with 2% cornmeal in 500 mL Erlenmeyer flasks containing 250 mL of the substrate [44] and grown in a stationary phase in the dark at 25 °C for 30 days.

### 4.3. Extraction and Purification Processes of Metabolites 1–11

The culture broth and mycelia were homogenized in a mixer with 350 mL of MeOH (1% NaCl). Then, the suspension was centrifuged for 40 min at 7000 rpm and 10 °C. The pellet was resuspended in 150 mL of a mixture of H_2_O:MeOH (45:55 *v*/*v*, 1% NaCl) and submitted to a second homogenization followed by centrifugation. Supernatants were collected, and MeOH was evaporated under reduced pressure to obtain an aqueous solution for the subsequent extraction (3 times) with ethyl acetate at native pH (=6.0). The organic phases were combined, dried with anhydrous Na_2_SO_4_, and evaporated under reduced pressure, yielding crude extract as brown oil (156.7 mg). The organic extract was purified by column chromatography (CC) on silica gel (40 cm × 1.5 cm i.d.) eluted with CHCl_3_/*i*-PrOH (95:5, *v*/*v*), originating 8 homogeneous fractions (A 3.7 mg, B 6.7 mg, C 43.3 mg, D 15.2 mg, E 9.1 mg, F 15.9 mg, G 2.3 mg, H 32.4 mg), the last of which was collected by eluting with methanol. Fraction C was purified by TLC on silica gel eluted with *n*-hexane/EtOAc (6:4, *v/v*) to give **2** (35.4 mg, white crystalline solid, R_f_ 0.59), **4** (2.0 mg white solid R_f_ 0.50), and **5** (1.2 mg yellowing oil, R_f_ 0.79). Fraction D was purified by TLC on silica gel eluted with *n*-hexane/EtOAc (1:1, *v/v*) to give **3** (5.2 mg, white crystalline solid, R_f_ 0.76), and a mixture of **6** and **7**, which was separated by reversed-phase TLC using H_2_O-EtOH (1:1, *v/v*) (2.5 and 3.1 mg, white amorphous solids, R_f_ 0.54 and 0.58, respectively), **9** (2.1 mg, homogeneous oil, R_f_ 0.48), and **8** (1.3 mg, yellowing oil, R_f_ 0.45). Fraction F was purified by TLC on silica gel eluted with CHCl_3_/*i*-PrOH (95:5, *v/v*), giving **10** (7.8 mg as colorless oil, R_f_ 0.42), **11** (1.5 mg as white amorphous solid, R_f_ 0.39), and **1** (5.4 mg, yellowing amorphous solid, R_f_ 0.37).

Diplopyrone C (**1**): yellowing amorphous solid; [α]^25^_D_ +36 (c 0.24); IR ν_max_: 3433, 1720, 1629, 1373, 1254, 1221 cm^−1^; UV λ_max_ nm (log ε) 203 (2.87); ^1^H and ^13^C NMR spectra: see Table 1; HR-ESIMS (+) *m*/*z*: 235.0395 [calcd. for C_10_H_12_KO_4_ 235.0373, M + K]^+^, 219.1860 [calcd. for C_10_H_12_NaO_4_ 219.1896, M + Na]^+^, 179.0721 [calcd. for C_10_H_11_O_3_ 179.0708 M-OH]^+^.

### 4.4. Insects Rearing and Oral Toxicity Bioassay

*Acyrthosiphon pisum* was reared on potted broad bean plants (*Vicia faba*) at 20 ± 1 °C, 75 ± 5% RH, and under a 16:8 h light:dark photoperiod, starting with insects originally collected from alfalfa plants in Eboli, southern Italy. In order to synchronize the aphid population, parthenogenetic adult females were placed on plants for 6 h, resulting in neonate nymphs with an age of 0–6 h that were used throughout the experiments.

The oral toxicity of sphaeropsidin A (**2**) on *A. pisum* was investigated using a standard basal diet previously developed for assays of test compounds [45]. The feeding system for the pea aphid was realized as described in [45] with minor modifications. Each experimental unit was a feeding system with 10 aphids; four replications per treatment were realized, each replicate consisting of three experimental units per treatment. A total of 120 aphids per treatment were used. In each feeding system, 300 µL of artificial diet containing **1** 3.6 mM, 7.2 mM, 14.4 mM, and 28.8 mM was dispensed; negative control was realized using the artificial diet only. The experiment was carried out under the rearing conditions described above. Briefly, on day 0, neonate nymphs were transferred to a freshly prepared diet sachet feeding apparatus. Mortality was recorded daily for one week, and dead nymphs were removed. The artificial diet was replaced every two days. The lethal concentrations of **1** resulting in 10%, 50%, and 90% aphid mortality (defined as LC_10_, LC_50_, and LC_90_) and the corresponding 95% confidence intervals were determined.

### 4.5. Statistical Analysis

Aphid survival curves were compared using Kaplan–Meier and log-rank analysis. The results obtained were analyzed using non-linear sigmoid curve fitting, and the activity of each treatment was evaluated on day 6 on the basis of dose–response concentrations; the goodness of fit to the curve model was evaluated on the basis of R^2^ values. Data were analyzed using Prism 6 (GraphPad Software Inc. version 6.0b, San Diego, CA, USA).

## Figures and Tables

**Table 1:** NMR data of diplopyrone C (**1**) in CDCl_3_ ^1,2^.

No.	δC	δH (*J* in Hz)	HMBC
**1**	-	-	
**2**	162.8	-	
**3**	122.9	6.17 (1H) br d (9.8)	C-2, C-5
**4**	144.3	7.03 (1H) dd (9.8, 5.4)	C-2, C-5, C-6
**5**	63.2	4.28 (1H) dd (5.4, 3.3)	C-3, C-4, C-6
**6**	76.9	5.40 (1H) dd (7.9, 3.3)	C-7, C-8
**7**	127.2	5.93 (1H) ddd (11.7, 7.9, 1.1)	C-9
**8**	132.4	5.63 (1H) ddd (11.7, 6.7, 1.1)	C-6, C-9, C-10
**9**	55.5	3.34 (1H) dd (6.7, 2.1)	C-8, C-7, C-10
**10**	56.7	3.00 (1H) dq (5.2, 2.1)	C-11
**11**	17.5	1.40 (3H) d (5.2)	C-9, C-8

^1^ The chemical shifts are in δ values (ppm). ^2^ 2D ^1^H, ^1^H (COSY) ^13^C, and ^1^H (HSQC) NMR experiments delineated the correlations of all the protons and the corresponding carbons. Numbering is according to that in Figure 1.

**Table 2:** Secondary metabolites identified in this work and previously reported as products of *Diplodia* spp.

Code	Name	Source	Ref.
**2**	Sphaeropsidin A	*D. corticola*, *D. sapinea*, *D. africana*, *D. quercivora*	[3,10,34,35,36,37]
**3**	Sphaeropsidin B	*D. corticola*, *D. sapinea*	[10,34,37]
**4**	Sphaeropsidin C	*D. corticola*, *D. sapinea*, *D. quercivora*	[10,34,36,37]
**5**	(*R*)-mellein	*D. africana*, *D. fraxini*, *D. mutila*, *D. seriata*, *D. sapinea*	[3,4,21,38]
**6**	(3*R*,4*R*)-4-hydroxymellein	*D. corticola*, *D. africana*, *D. sapinea*	[3,10,21]
**7**	(3*S*,4*R*)-4-hydroxymellein	*D. corticola*, *D. africana*, *D. sapinea*	[3,10,21]
**8**	Sapinofuranone B	*D. corticola*	[10]
**9**	Pinofuranoxin A	*D. sapinea*	[23]
**10**	Diplobifuranylone B	*D. corticola*	[10]
**11**	Tyrosol	*D. fraxini*, *D. mutila*	[4,38]

## Data Availability

The data that support the findings of this study are available from the corresponding author upon reasonable request.

## Supplementary Materials

The following supporting information can be downloaded at: https://www.mdpi.com/article/10.3390/molecules27072327/s1, Figure S1: ^1^H NMR spectrum of diplopyrone C (**1**) recorded in CDCl_3_ at 400 MHz; Figure S2: ^13^C NMR spectrum of diplopyrone (**1**) recorded in CDCl_3_ at 100 MHz; Figure S3: ^1^H,^1^H COSY spectrum of diplopyrone C (**1**) recorded in CDCl_3_ at 400 MHz; Figure S4: HSQC spectrum of diplopyrone C (**1**) recorded in CDCl_3_ at 400 MHz; Figure S5: HMBC spectrum of diplopyrone C (**1**) recorded in CDCl_3_ at 400 MHz; Figure S6: NOESY spectrum of diplopyrone C (1) recorded in CDCl_3_ at 400 MHz; Figure S7: IR spectrum of diplopyrone C (**1**); Figure S8: HRESI MS spectrum of diplopyrone C (**1**) recorded in positive mode; Figure S9: ^1^H NMR spectrum of sphaeropsidin A (**2**) recorded in CDCl_3_ at 400 MHz; Figure S10: ^1^H NMR spectrum of sphaeropsidin B (**3**) recorded in CDCl_3_ at 400 MHz; Figure S11: ^1^H NMR spectrum of sphaeropsidin C (**4**) recorded in CDCl_3_ at 400 MHz; Figure S12: ^1^H NMR spectrum of (3*R*)-mellein (**5**) recorded at 400 MHz in CDCl3; Figure S13: ^1^H NMR spectrum of (3*R*,4*R*)-4-hydroxymellein (**6**) recorded at 400 MHz in CDCl_3_; Figure S14: ^1^H NMR spectrum of (3*R*,4*S*)-4-hydroxymellein (**7**) recorded at 400 MHz in CDCl_3_; Figure S15: ^1^H NMR spectrum sapinofuranone B (**8**) recorded at 400 MHz in CDCl_3_; Figure S16: ^1^H NMR spectrum of pinofuranoxin A (**9**) recorded at 400 MHz in CDCl_3_; Figure S17: ^1^H NMR spectrum of diplobifuranylone B (**10**) recorded at 400 MHz in CDCl_3_; Figure S18: ^1^H NMR spectrum tyrosol (**11**) recorded at 400 MHz in CDCl_3_.
