# Supplementary material for: Secondary Metabolites, including a New 5,6-Dihydropyran-2-One, Produced by the Fungus Diplodia corticola. Aphicidal Activity of the Main Metabolite, Sphaeropsidin A
Source: Molecules. 2022 Apr 4;27(7):2327. doi: 10.3390/molecules27072327 (PMC9000672; doi:10.3390/molecules27072327)
Supplement: Supplementary file 1 [file molecules-27-02327-s001.zip › molecules-1638243-supplementary.pdf]

Secondary Metabolites, Including a New 5,6-Dihydropyran-2-one, Produced by the Fungus *Diplodia corticola*. Aphicidal Activity of the Main Metabolite, Sphaeropsidin A

Maria Michela Salvatore[1,2a], Ilaria Di Lelio[3a], Marina DellaGreca[1], Rosario Nicoletti[3,4], Francesco Salvatore[1], Elia Russo[3], Gennaro Volpe[3], Andrea Becchimanzi[3], Alla Eddine Mahamedi[5], Akila Berraf-Tebbal[6], and Anna Andolfi[1,7*]

[1] Department of Chemical Sciences, University of Naples Federico II, 80126 Naples, Italy; mariamichela.salvatore@unina.it (M.M.S.); dellagre@unina.it (M.D.); frsalvat@unina.it (F.S.)
[2] Institute for Sustainable Plant Protection, National Research Council, 80055 Portici, Italy
[3] Department of Agriculture, University of Naples Federico II, 80055 Portici, Italy; ilaria.dilelio@unina.it (I.D.L.); rosario.nicoletti@crea.gov.it (R.N.); elia.russo@unina.it (E.R.); gennaro.volpe2@unina.it (G.V.); andrea.becchimanzi@unina.it (A.B.)
[4] Council for Agricultural Research and Economics, Research Center for Olive, Fruit, and Citrus Crops,
81100 Caserta, Italy
[5] Department of Biology, Faculty of Natural Sciences, Life and Earth Sciences, University of Ghardaia,
47000 Ghardaia, Algeria; aladin1342@yahoo.com
[6] Mendeleum-Institute of Genetics, Faculty of Horticulture, Mendel University in Brno, 69144 Lednice, Czech Republic; berraf.a@hotmail.fr
[7] BAT Center-Interuniversity Center for Studies on Bioinspired Agro-Environmental Technology, University of Naples Federico II, 80055 Portici, Italy
* Correspondence: andolfi@unina.it
† These authors contributed equally to this work.

Contents

Figure S1. ^{1}H NMR spectrum of diplopyrone C (**1**) recorded in $CDCl_3$ at 400 MHz

Figure S2. ^{13}C NMR spectrum of diplopyrone (**1**) recorded in $CDCl_3$ at 100 MHz

Figure S3. ^{1}H,^{1}H COSY spectrum of diplopyrone C (**1**) recorded in $CDCl_3$ at 400 MHz

Figure S4. HSQC spectrum of diplopyrone C (**1**) recorded in $CDCl_3$ at 400 MHz

Figure S5. HMBC spectrum of diplopyrone C (**1**) recorded in $CDCl_3$ at 400 MHz

Figure S6. NOESY spectrum of diplopyrone C (**1**) recorded in $CDCl_3$ at 400 MHz

Figure S7. IR spectrum of diplopyrone C (**1**)

Figure S8. HRESI MS spectrum of diplopyrone C (**1**) recorded in positive mode

Figure S9. ^{1}H NMR spectrum of sphaeropsidin A (**2**) recorded in $CDCl_3$ at 400 MHz

Figure S10. ^{1}H NMR spectrum of sphaeropsidin B (**3**) recorded in $CDCl_3$ at 400 MHz

Figure S11. ^{1}H NMR spectrum of sphaeropsidin C (**4**) recorded in $CDCl_3$ at 400 MHz

Figure S12. ^{1}H NMR spectrum of (3*R*)-mellein (**5**) recorded at 400 MHz in $CDCl_3$

Figure S13. ^{1}H NMR spectrum of (3*R*,*4R*)-4-hydroxymellein (**6**) recorded at 400 MHz in $CDCl_3$

Figure S14. ^{1}H NMR spectrum of (3*R*,*4S*)-4-hydroxymellein (**7**) recorded at 400 MHz in $CDCl_3$

Figure S15. ^{1}H NMR spectrum sapinofuranone B (**8**) recorded at 400 MHz in $CDCl_3$

Figure S16. ^{1}H NMR spectrum of pinofuranoxin A (**9**) recorded at 400 MHz in $CDCl_3$

Figure S17. ^{1}H NMR spectrum of diplobifuranylone B (**10**) recorded in $CDCl_3$ at 400 MHz

Figure S18. ^{1}H NMR spectrum tyrosol (**11**) recorded at 400 MHz in $CDCl_3$

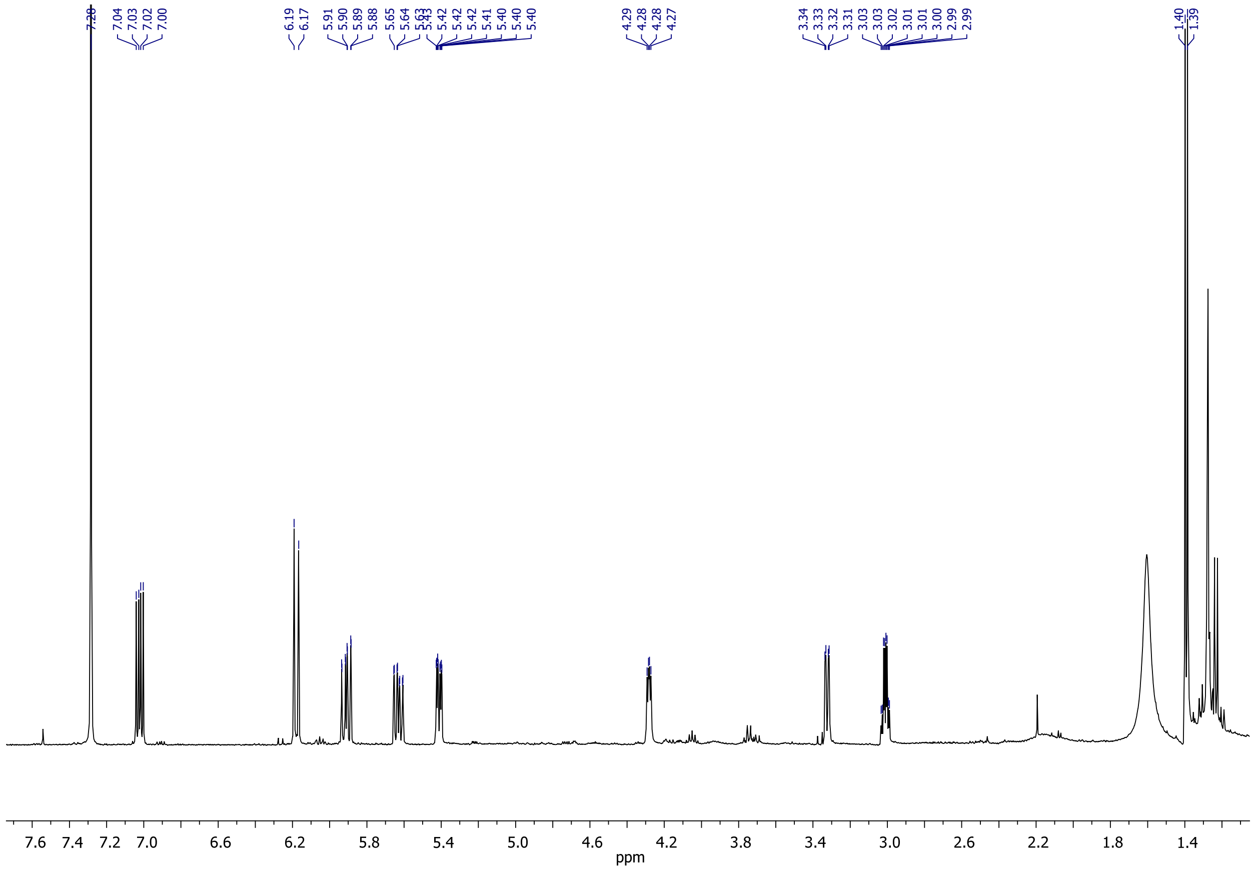

Figure S1. ^1H NMR spectrum of diplopyrone C (**1**) recorded in $CDCl_3$ at 400 MHz.

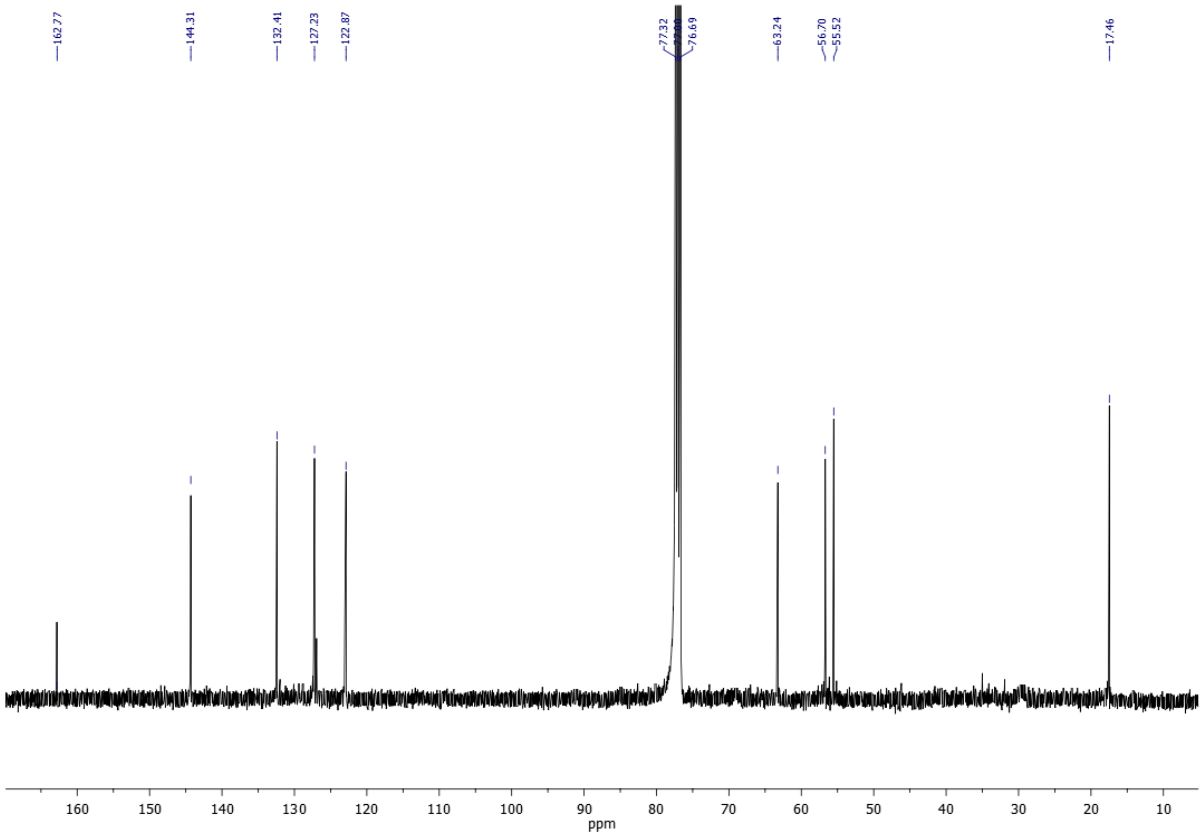

Figure S2. ^{13}C NMR spectrum of diplopyrone (**1**) recorded in $CDCl_3$ at 100 MHz.

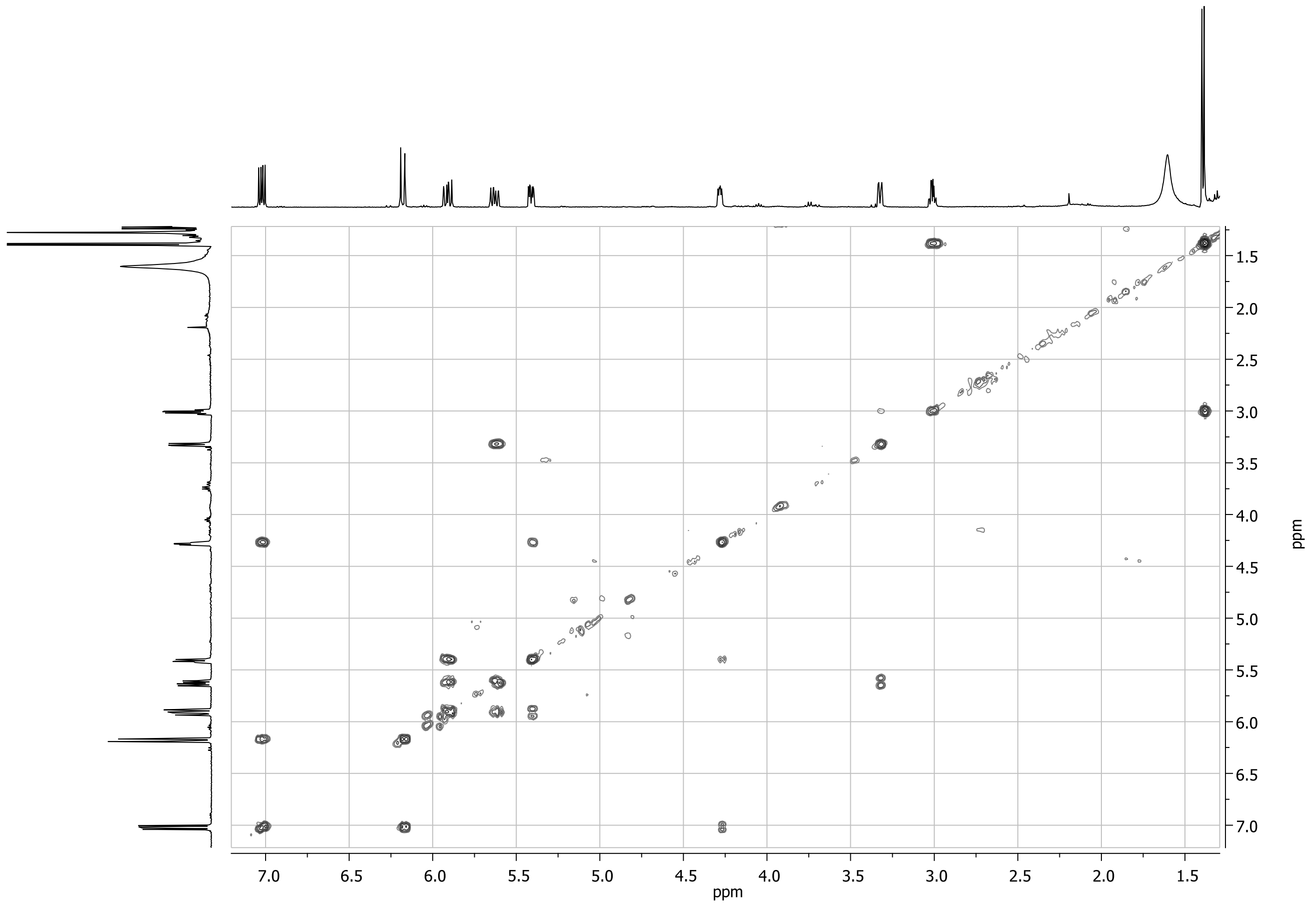

Figure S3. $^{1}H,^{1}H$ COSY spectrum of diplopyrone C (**1**) recorded in $CDCl_3$ at 400 MHz.

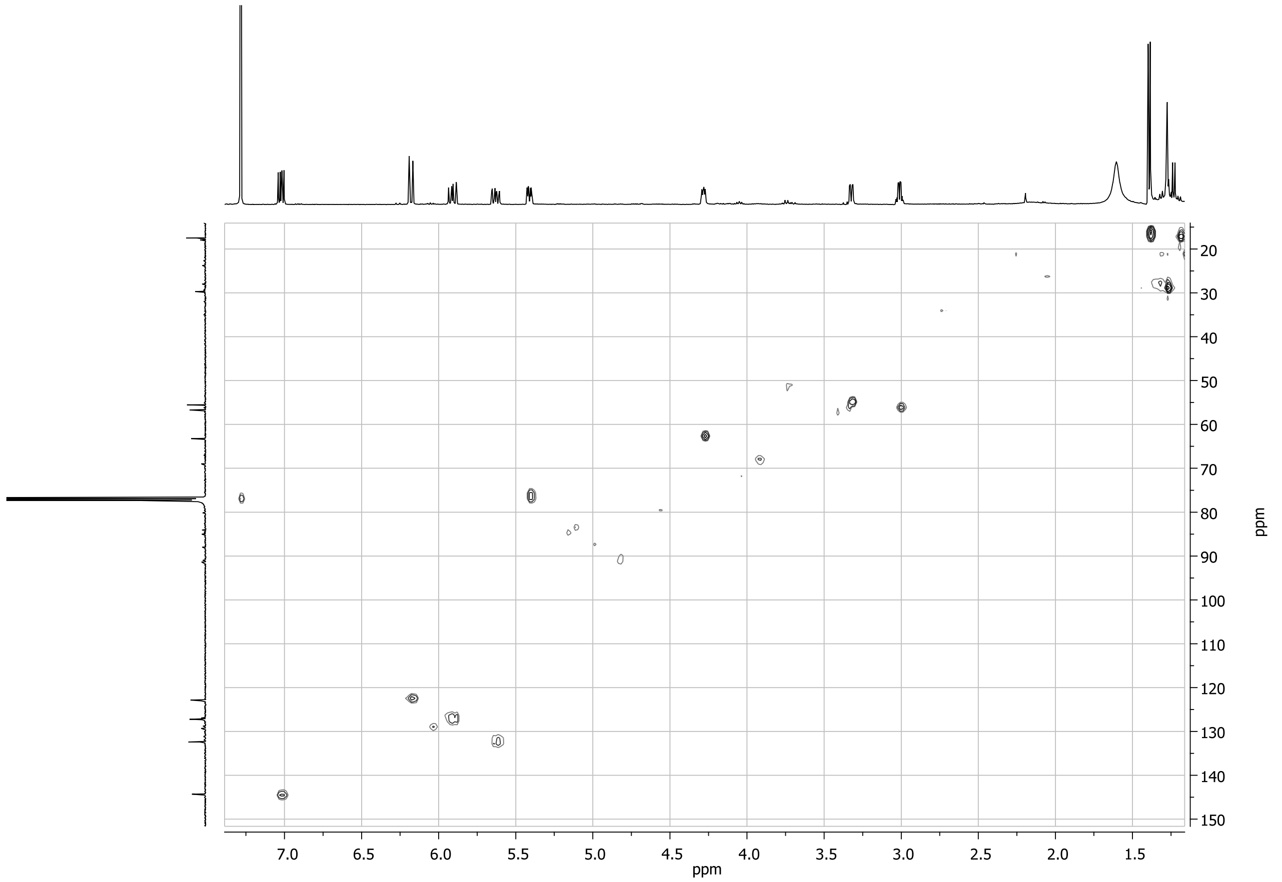

Figure S4. HSQC spectrum of diplopyrone C (**1**) recorded in $CDCl_3$ at 400 MHz.

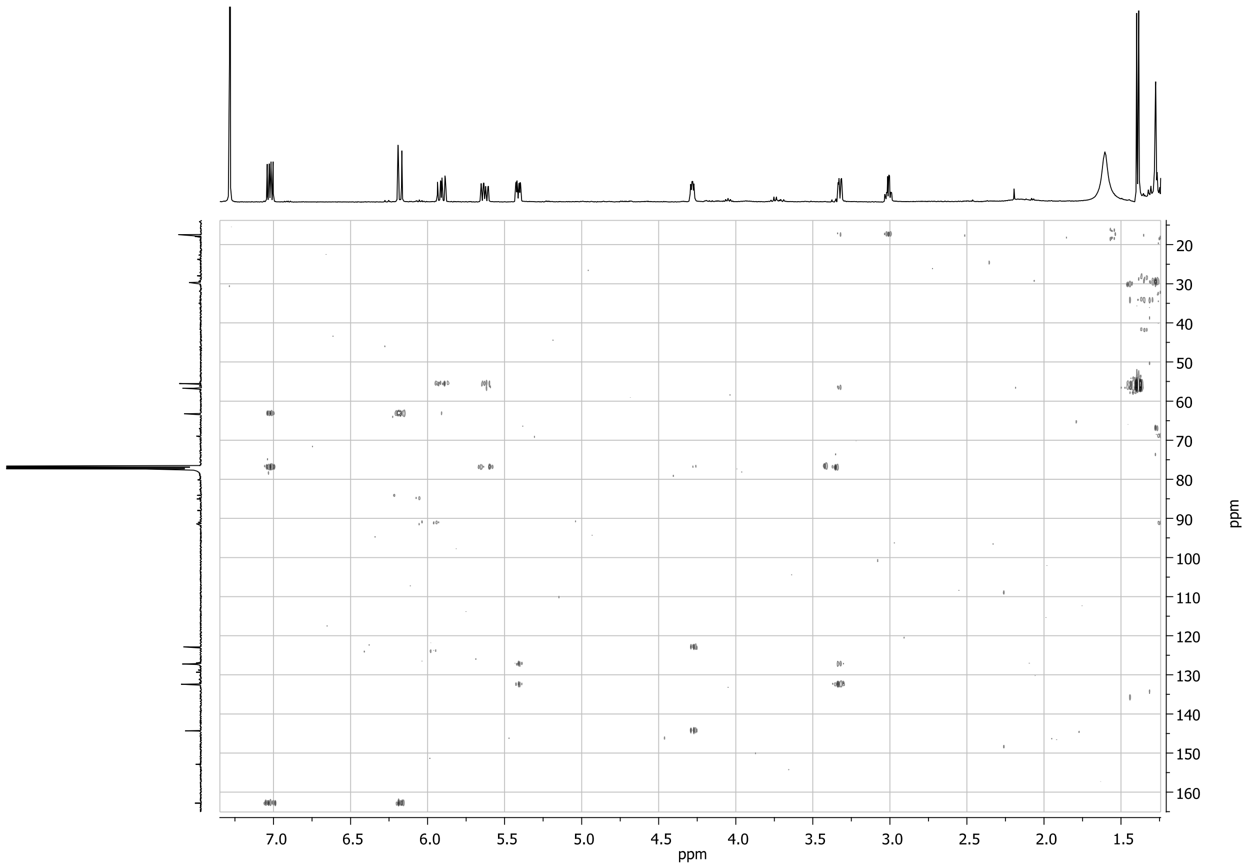

Figure S5. HMBC spectrum of diplopyrone C (**1**) recorded in $CDCl_3$ at 400 MHz.

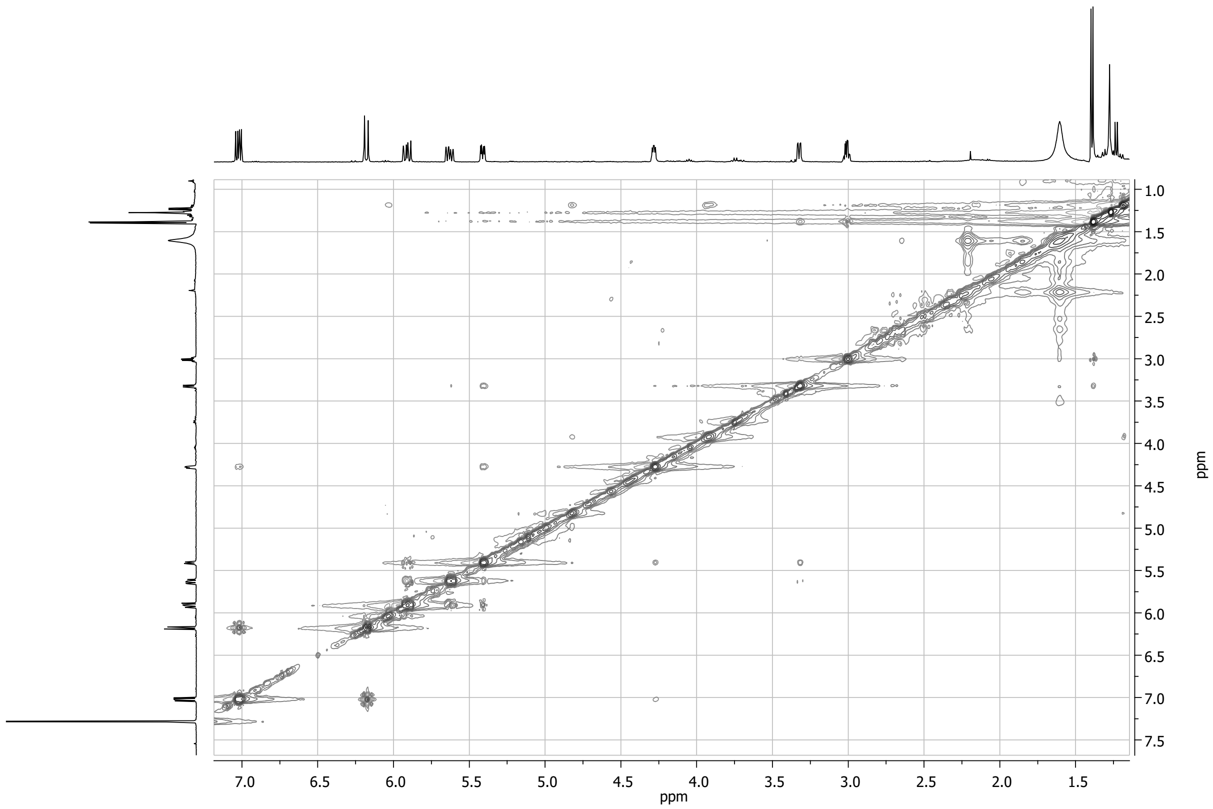

Figure S6. Noesy spectrum of diplopyrone C (**1**) recorded in $CDCl_3$ at 400 MHz.

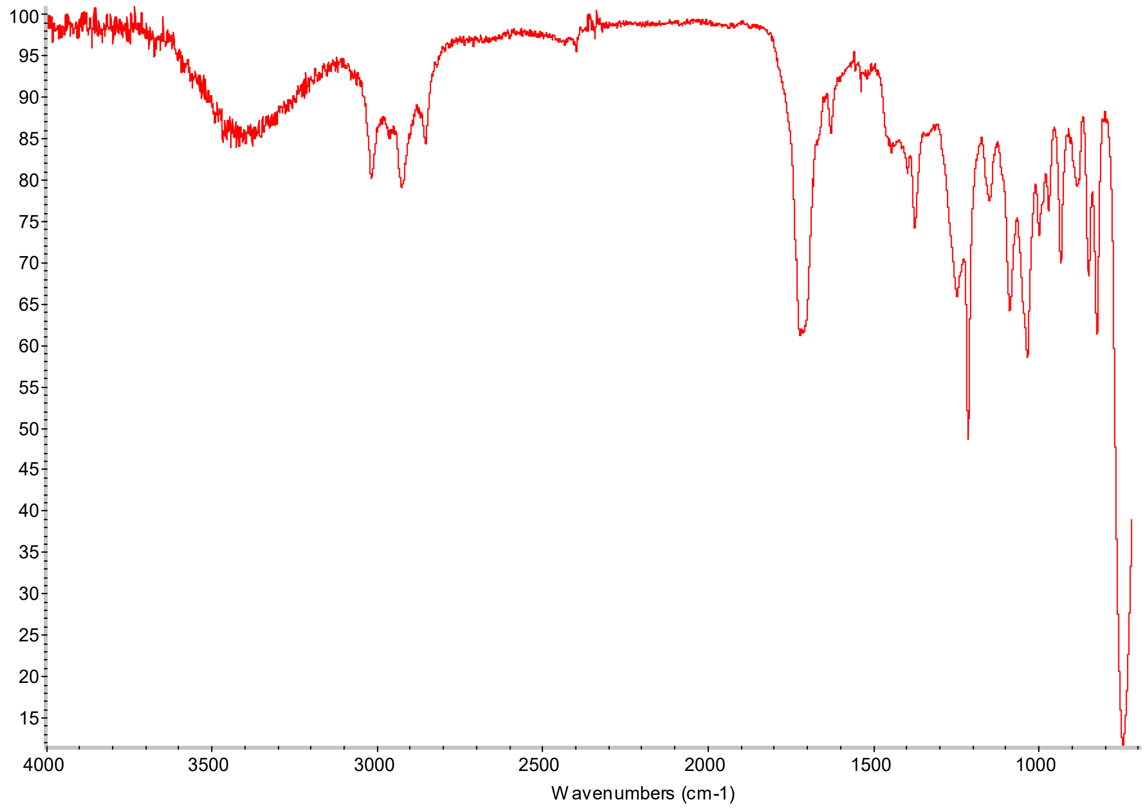

Figure S7. IR spectrum of diplopyrone C (**1**).

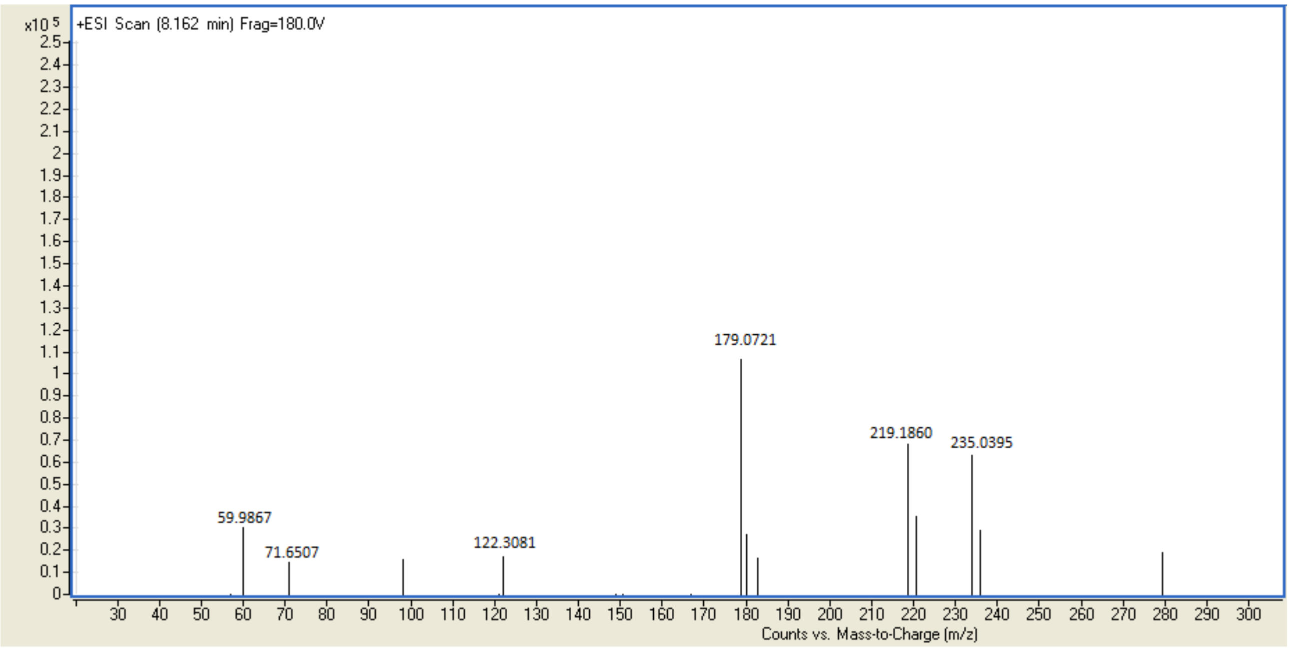

Figure S8. HRESI MS spectrum of diplopyrone C (**1**) recorded in positive mode.

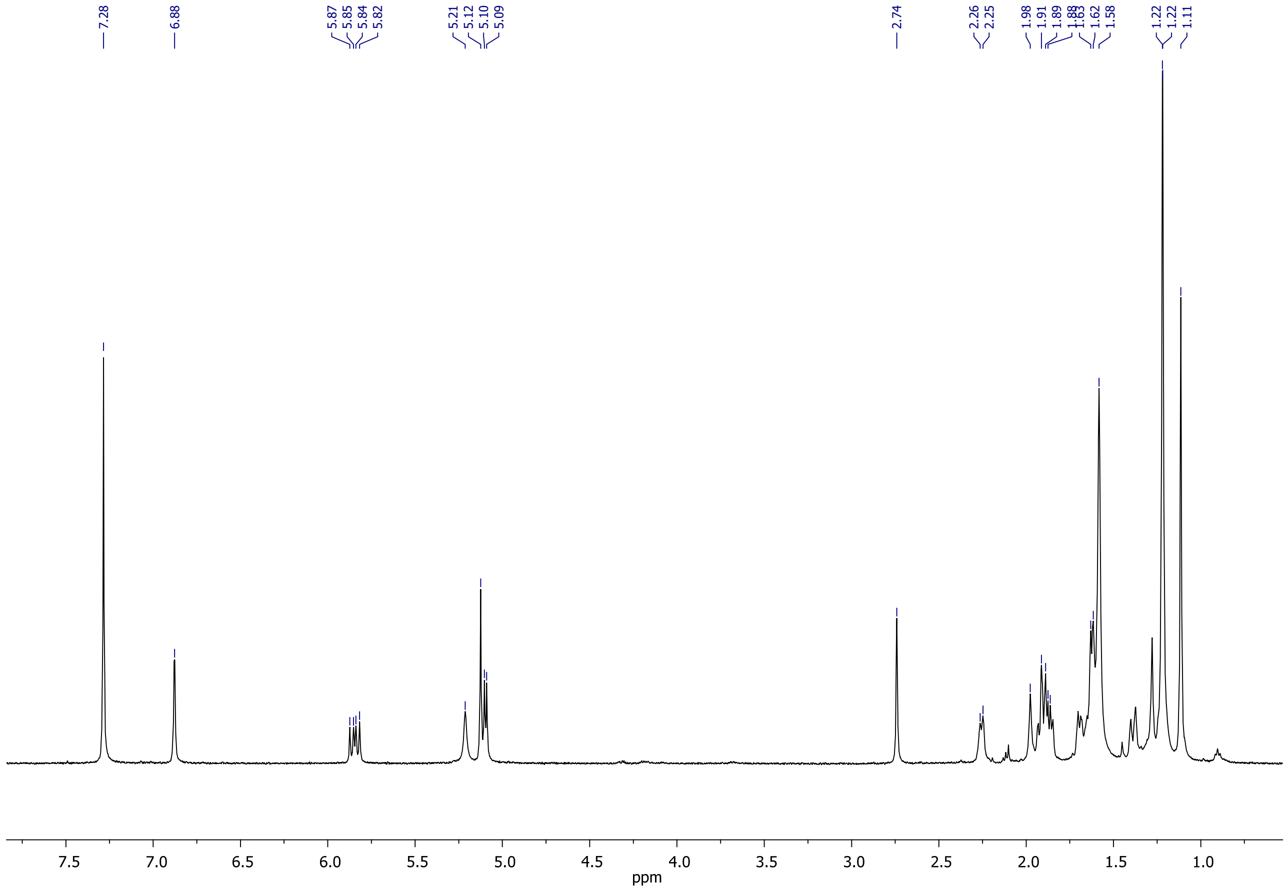

Figure S9. ^{1}H NMR spectrum of sphaeropsidin A (**2**) recorded in $CDCl_3$ at 400 MHz.

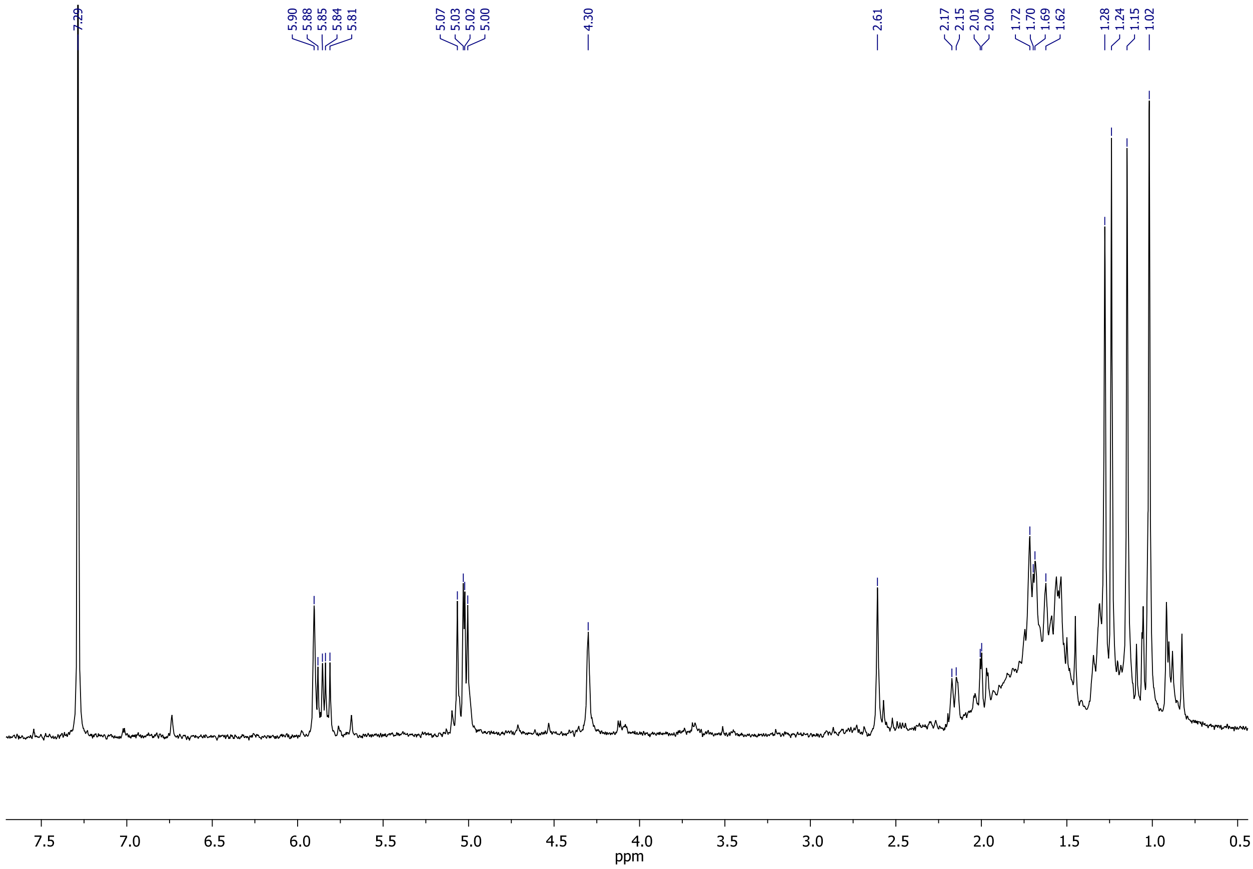

Figure S10. ^{1}H NMR spectrum of sphaeropsidin B (**3**) recorded in $CDCl_3$ at 400 MHz.

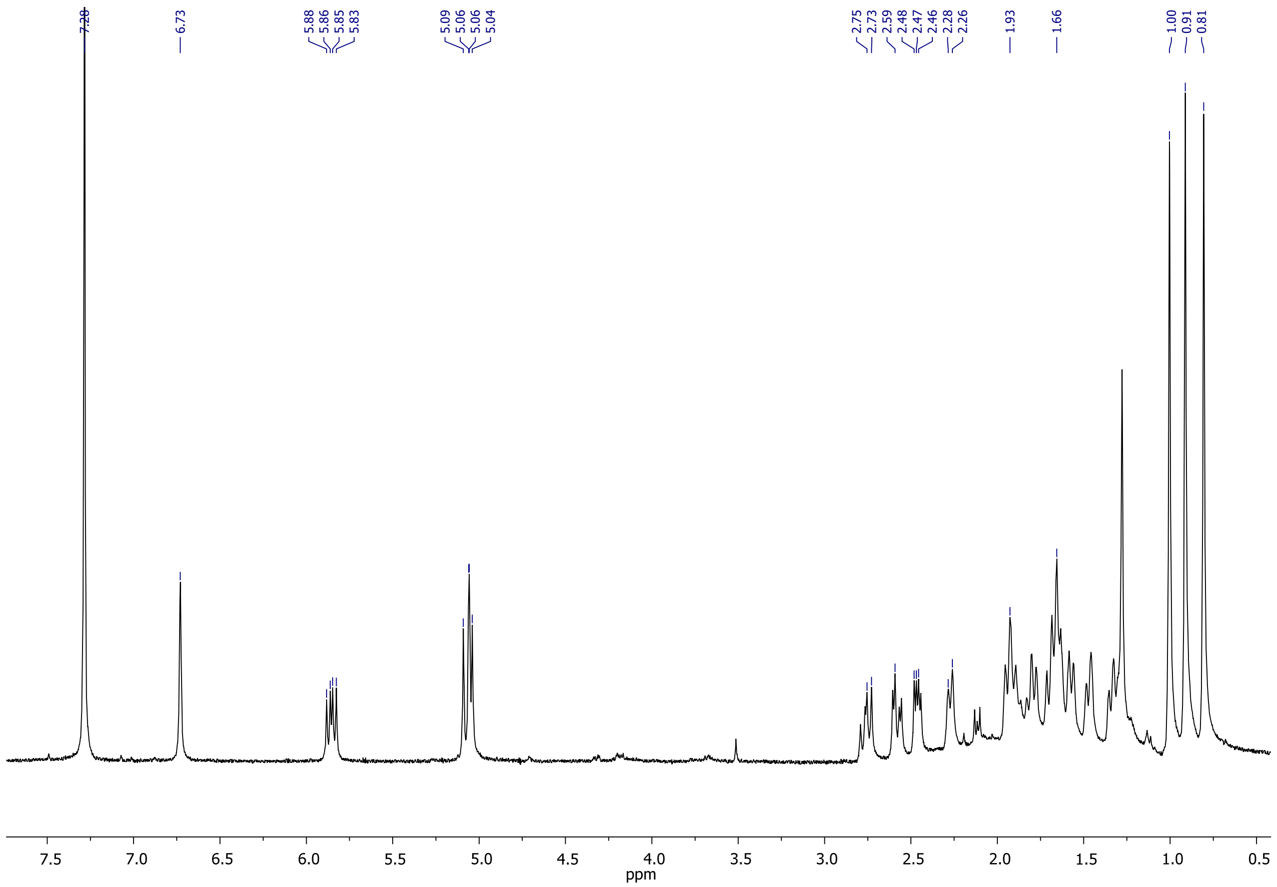

Figure S11. ^{1}H NMR spectrum of sphaeropsidin C (**4**) recorded in $CDCl_3$ at 400 MHz.

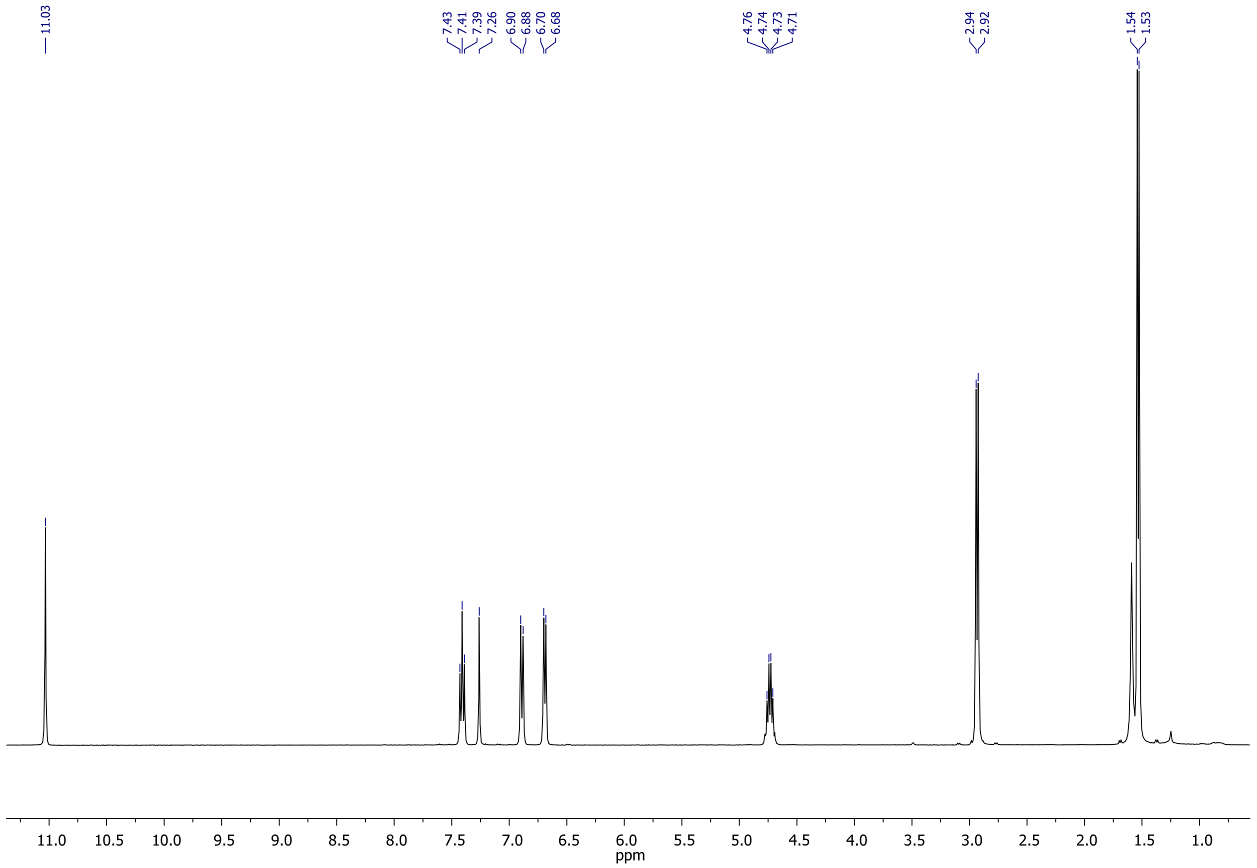

Figure S12. ^{1}H NMR spectrum of (3*R*)-mellein (**5**) recorded at 400 MHz in $CDCl_3$.

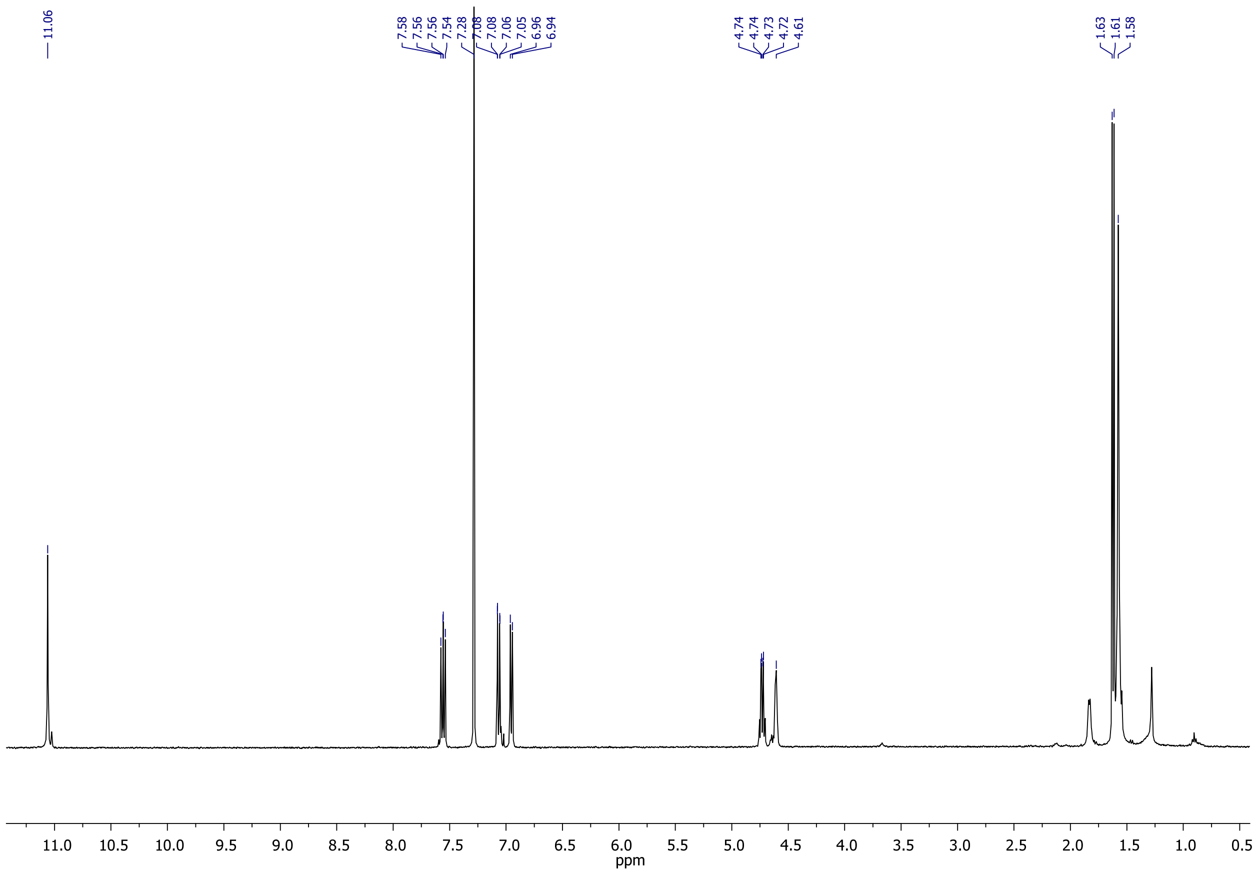

Figure S13. ^{1}H NMR spectrum of (3*R*,4*R*)-4-hydroxymellein (**6**) recorded at 400 MHz in $CDCl_3$.

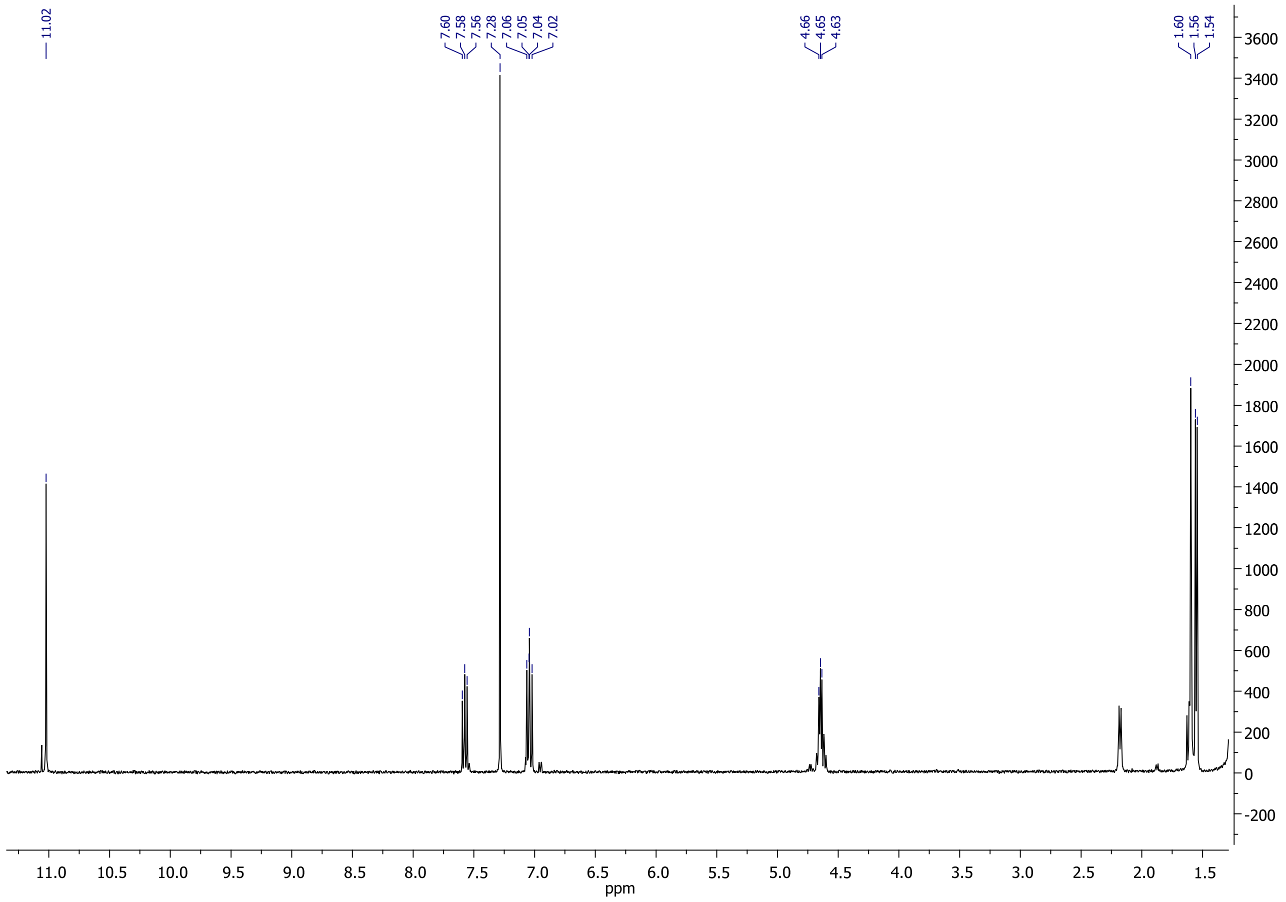

Figure S14. ^{1}H NMR spectrum of (3*R*,4*S*)-4-hydroxymellein (**7**) recorded at 400 MHz in $CDCl_3$.

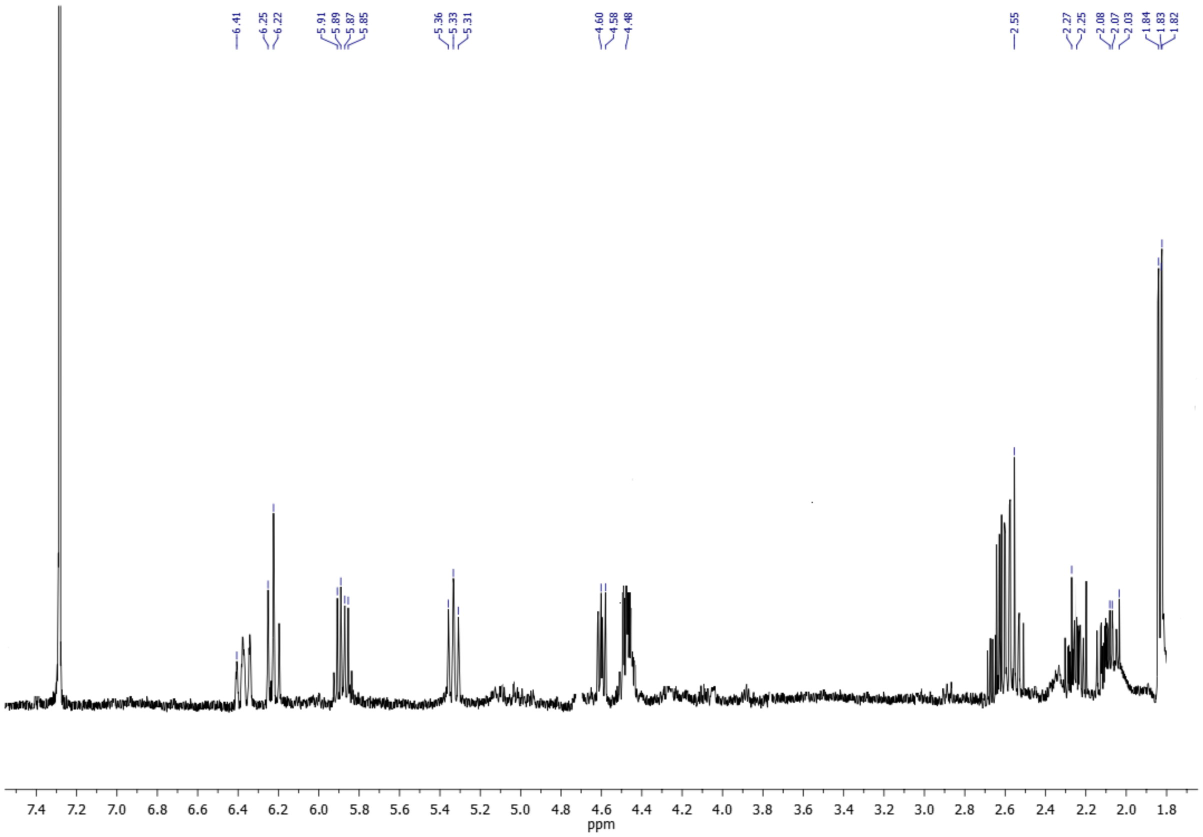

Figure S15. ^{1}H NMR spectrum sapinofuranone B (**8**) recorded at 400 MHz in $CDCl_3$.

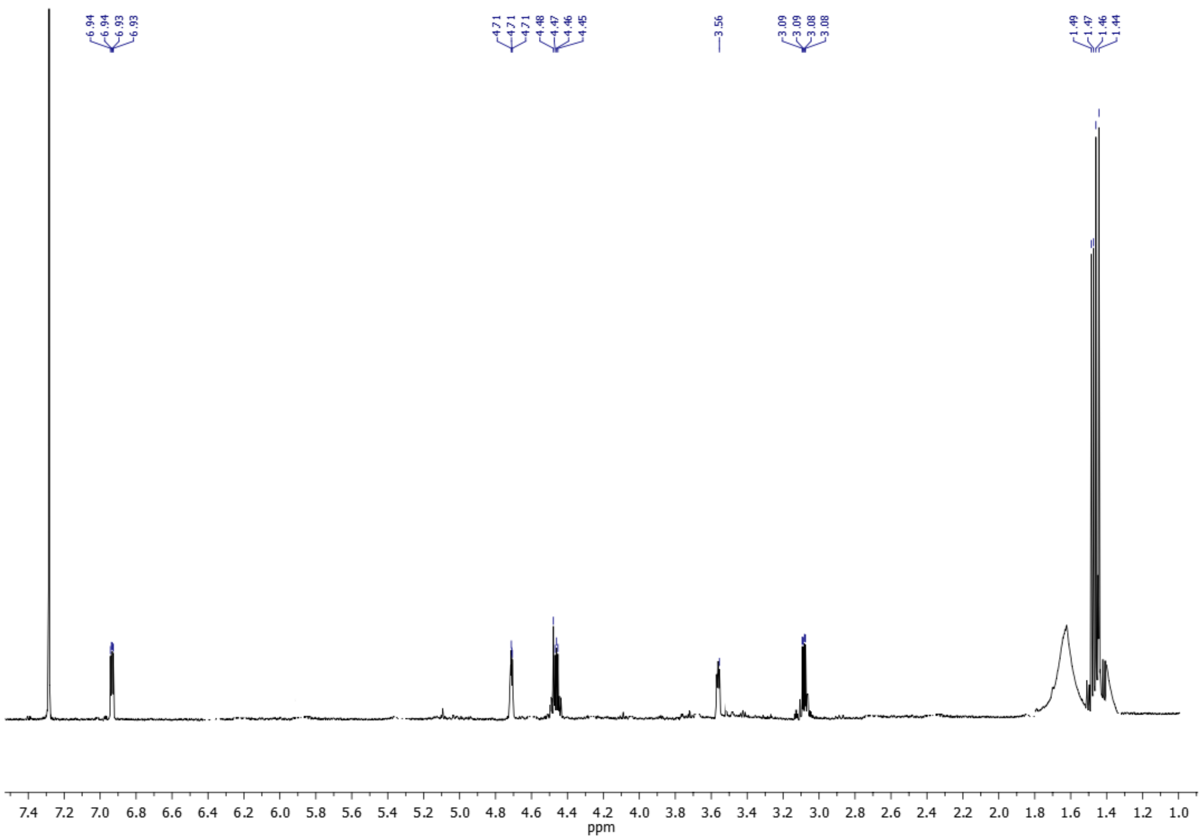

Figure S16. ^{1}H NMR spectrum of pinofuranoxin A (**9**) recorded at 400 MHz in $CDCl_3$.

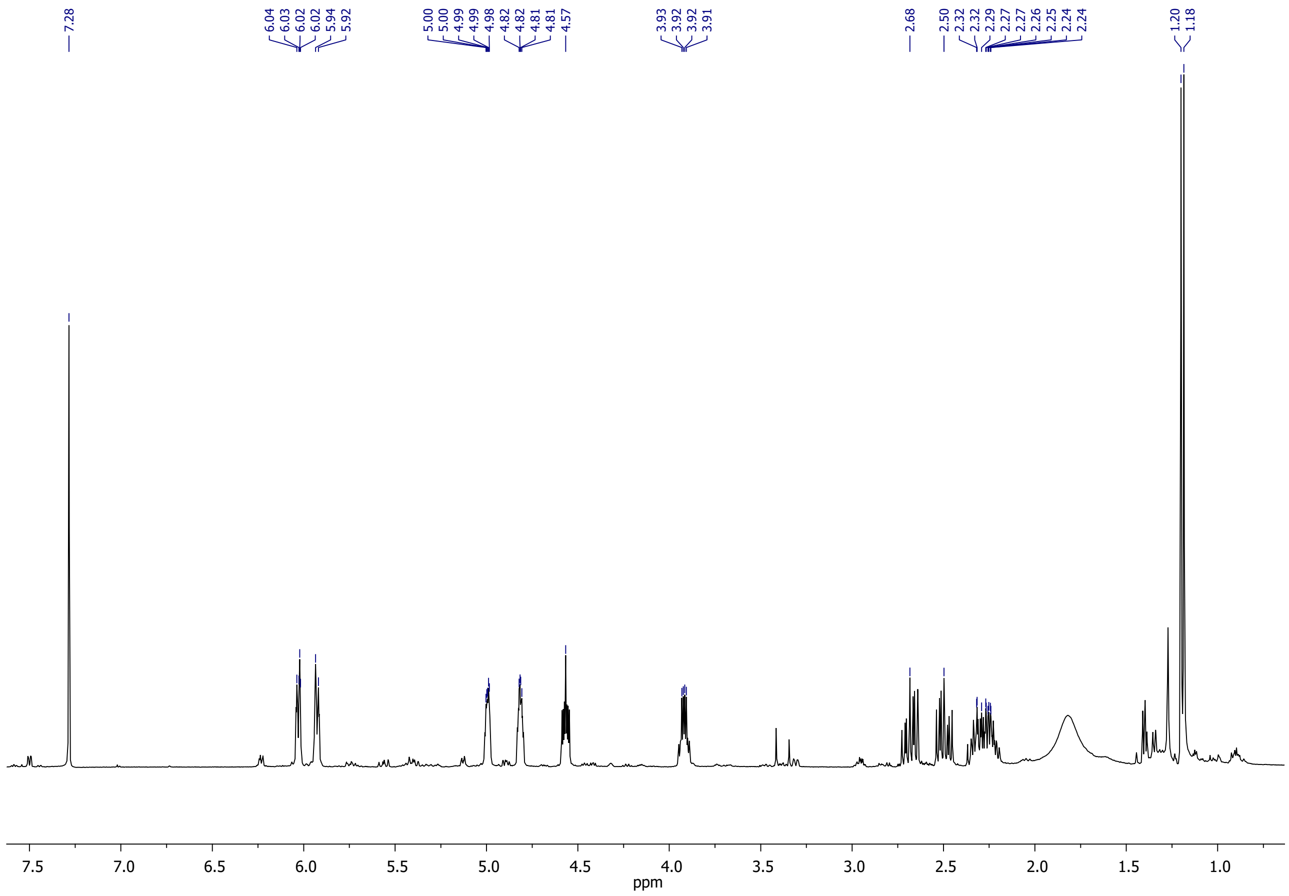

Figure S17. ^{1}H NMR spectrum of diplobifuranylone B (**10**) recorded in $CDCl_3$ at 400 MHz.

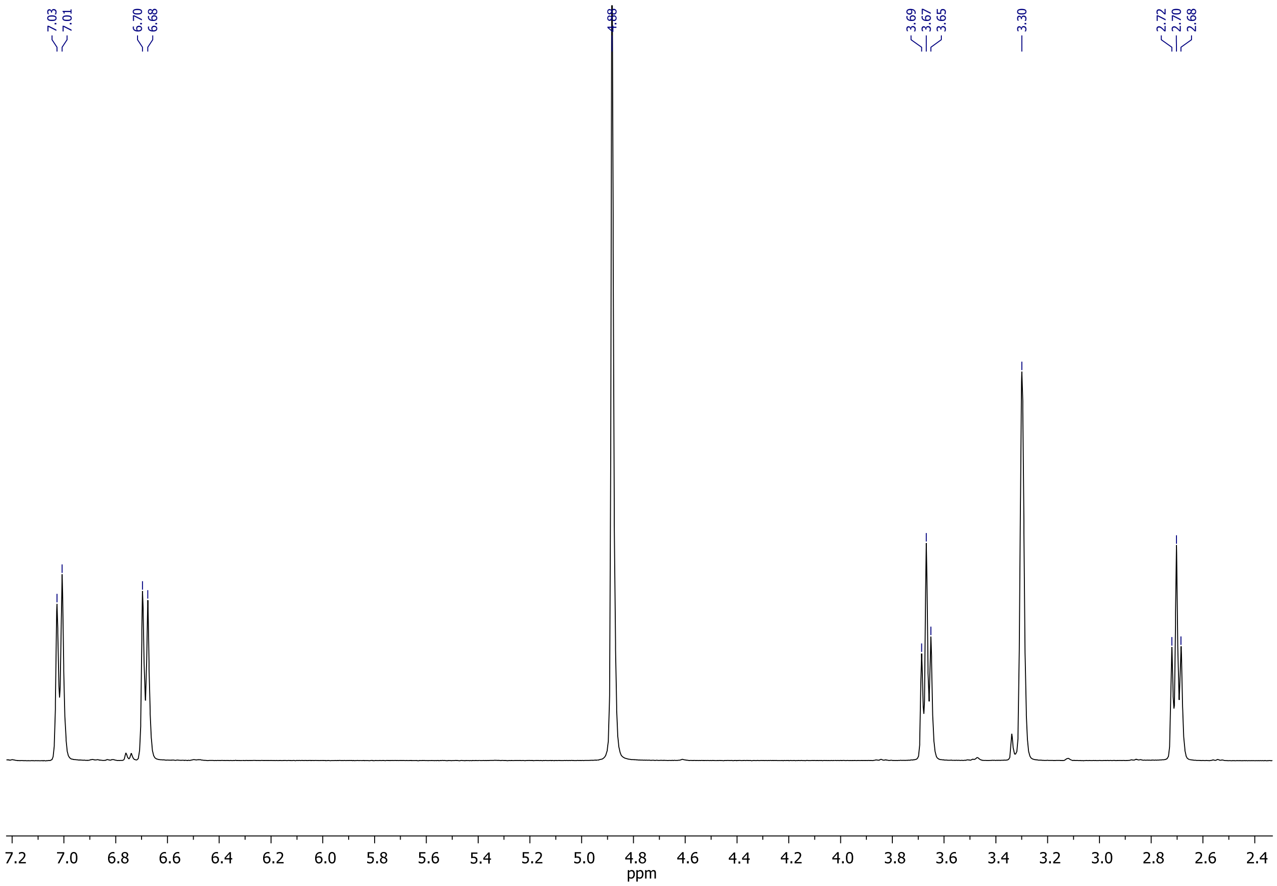

Figure S18. ^{1}H NMR spectrum tyrosol (**11**) recorded at 400 MHz in $CDCl_3$.
